# Comparison of Ketogenic Diets with and without Ketone Salts versus a Low-Fat Diet: Liver Fat Responses in Overweight Adults

**DOI:** 10.3390/nu13030966

**Published:** 2021-03-17

**Authors:** Christopher D. Crabtree, Madison L. Kackley, Alexandru Buga, Brandon Fell, Richard A. LaFountain, Parker N. Hyde, Teryn N. Sapper, William J. Kraemer, Debbie Scandling, Orlando P. Simonetti, Jeff S. Volek

**Affiliations:** 1 Department of Human Sciences, The Ohio State University, Columbus, OH 43201, USA; crabtree.223@osu.edu (C.D.C.); kackley.19@osu.edu (M.L.K.); buga.1@osu.edu (A.B.); brandon.fell@virtahealth.com (B.F.); lafountainrich@gmail.com (R.A.L.); parker.hyde@ung.edu (P.N.H.); bedell.387@osu.edu (T.N.S.); kraemer.44@osu.edu (W.J.K.); 2 Dorothy M. Davis Heart & Lung Research Institute, The Ohio State University, Columbus, OH 43210, USA; debbie.scandling@osumc.edu (D.S.); orlando.simonetti@osumc.edu (O.P.S.); 3 Departments of Radiology and Internal Medicine, The Ohio State University, Columbus, OH 43210, USA

**Keywords:** NAFLD, liver fat, low carbohydrate, ketogenic diet, exogenous ketones

## Abstract

Ketogenic diets (KDs) often contain high levels of saturated fat, which may increase liver fat, but the lower carbohydrate intake may have the opposite effect. Using a controlled feeding design, we compared liver fat responses to a hypocaloric KD with a placebo (PL) versus an energy-matched low-fat diet (LFD) in overweight adults. We also examined the added effect of a ketone supplement (KS). Overweight adults were randomized to a 6-week KD (KD + PL) or a KD with KS (KD + KS); an LFD group was recruited separately. All diets were estimated to provide 75% of energy expenditure. Weight loss was similar between groups (*p* > 0.05). Liver fat assessed by magnetic resonance imaging decreased after 6 week (*p* = 0.004) with no group differences (*p* > 0.05). A subset with nonalcoholic fatty liver disease (NAFLD) (liver fat > 5%, *n* = 12) showed a greater reduction in liver fat, but no group differences. In KD participants with NAFLD, 92% of the variability in change in liver fat was explained by baseline liver fat (*p* < 0.001). A short-term hypocaloric KD high in saturated fat does not adversely impact liver health and is not impacted by exogenous ketones. Hypocaloric low-fat and KDs can both be used in the short-term to significantly reduce liver fat in individuals with NAFLD.

## 1. Introduction

Excessive adiposity affects a third of the global population with projections that by 2030 half of men and women will be obese [1,2]. Obesity is strongly associated with multiple conditions such as cardiovascular disease, type 2 diabetes, hypertension, and non-alcoholic fatty liver disease (NAFLD) [3]. As a response to the growing rate of obesity and associated co-morbidities, many people have turned to novel dietary formulations such as the ketogenic diet (KD) in order to lose weight [4]. This growing cultural shift in eating habits toward a preference for consuming dietary fats, including saturated fats, has led to questions regarding the safety of KD in obese populations and others at increased risk of NAFLD [5]. NAFLD is a progressive liver disease characterized by increased accumulation of fat in the liver (>5% fat), that if left untreated can lead to non-alcoholic steatohepatitis, fibrosis, cirrhosis, and liver failure [6]. In overweight subjects, over-consumption of saturated fat induces a greater increase of hepatic triglycerides compared to unsaturated fat and simple sugars in the context of a non-KD [7]. In mouse models, a high saturated fat KD has been shown to adversely impact liver health in some [8,9,10], but not all [11] studies, but these results are difficult to interpret due to variations in KD formulation (especially protein) and species differences between mice and humans in hepatic glucose and lipid metabolism. Increased levels of aminotransferases in response to a therapeutic KD has been reported in children with epilepsy [12]; however, this adverse effect may be attributed to valproate medication, which is commonly prescribed in this population [13].

From a dietary-metabolic perspective, a low total and saturated fat/high-carbohydrate diet (LFD) could be effective in managing liver fat storage by limiting exogenous sources of fat; however, the higher carbohydrate intake may not favor the net loss of liver fat due to increased de novo lipogenesis (DNL) and reduced fatty acid oxidation and/or ketogenesis. In contrast, low-carbohydrate/high-fat KD induce significantly increased rates of whole-body fatty acid oxidation and hepatic ketogenesis [14,15,16]. Thus, both LFD and KD have been demonstrated to reduce liver fat [17,18,19,20,21], but whether one approach is superior to the other in terms of effectiveness, when matched for energy, is less understood. Hypocaloric KDs have been shown to rapidly decrease liver fat [14,16], and in the short-term (3–14 days) outperform LFDs despite being matched for energy content and weight loss [22,23]. Over longer periods (2–6 months), a KD that results in significant weight loss may result in a greater decrease in liver fat than an LFD [24], but when weight loss is similar between KDs and LFDs there appears to be no difference in liver fat responses [18,22]. Since previous comparison studies did not involve controlled feeding designs over the entire duration of the intervention [18,22,23,24], compliance to dietary protocols is questionable. Furthermore, failure to achieve nutritional ketosis in the low-carbohydrate arms of prior studies [18,24] may limit the utility of their results because partitioning of hepatic fatty acids into ketogenesis is a primary mechanism contributing to decreased liver fat [16]. To address these limitations, we performed a 6-week controlled feeding study in overweight/obese subjects who consumed a precisely defined, equally hypocaloric KD or LFD.

Exogenous ketone supplements can augment circulating concentration of ketones and have been theorized as beneficial for NAFLD pathology, possibly even without adherence to KD [25]. Metabolically, ketones inhibit adipose tissue lipolysis [26], and could thereby positively impact fat balance by decreasing hepatic fatty acid delivery. Exogenous ketones have also been shown to decrease hepatic gluconeogenesis and increase peripheral glucose uptake [27] which could translate into improved liver health by decreasing substrate for de novo lipogenesis.

The primary objective of this study was to examine the effect of a well-balanced hypocaloric KD, with and without exogenous KS supplementation, compared to a hypocaloric LFD on liver fat storage following a 6-week controlled-feeding intervention. We hypothesized that the differing dietary approaches would produce similar reductions in liver fat, especially in those with NAFLD, while not inducing further negative hepatic outcomes from increased saturated fat consumption. The primary outcome was composite liver fat fraction assessed by magnetic resonance imaging (MRI); fat content was also assessed in individual liver segments to address potential regional anatomical differences [28]. Secondary outcomes included changes in liver enzymes and other biomarkers associated with liver health.

## 2. Materials and Methods

### 2.1. Study Design

This project was part of a larger study that involved a 2-arm randomized controlled feeding trial designed to assess the effects of a KD with an exogenous ketone salt supplement (KD + KS) and a KD with placebo (KD + PL) on multiple outcomes including body composition and measures of cardiometabolic risk [29]. Here, we focus liver fat responses and related biomarkers. Twenty-eight participants were enrolled and randomized 1:1 to either the KD + PL or KD + KS group. Two participants in the KD + KS and one participant in the KD + PL did not complete the study. A body mass index (BMI) and age matched group of participants (*n* = 12) were assigned post-hoc to an energy- and protein-matched LFD. In our experience, individuals often favor either a low-carbohydrate or low-fat approach. Therefore, considering the high level of commitment required to comply with the feeding intervention, subjects were randomly assigned to the KD + KS and KD + PL groups, but we recruited separately for the LFD to avoid forcing subjects into a non-preferred eating pattern that could jeopardize compliance.

### 2.2. Human Subjects

We recruited participants classified as either overweight (25.0 ≤ BMI ≤ 29.9 kg/m^2^) or Class 1 obese (30.0 ≤ BMI ≤ 34.9 kg/m^2^), 21–65 years of age with an expressed goal to lose weight. Interested participants completed a food frequency survey, medical history, physical activity questionnaire, MRI screening, and menstrual history to determine eligibility. Participants were excluded if they had chronic diseases (e.g., diabetes, cancer, heart disease, etc.), endocrine dysfunction, current smoking or drug use, alcoholism, epilepsy, chronic headaches, pregnancy, use of antibiotic medication, >10% weight loss in prior six months, or current use of a KD. Consented subjects were instructed to maintain their regular exercise habits throughout the study. Baseline physical activity questionnaires were quantified on the basis of activity intensity and time spent per week (METs* minutes of activity per week) and compared between groups, showing no significant differences (Table 1). Biweekly physical activity assessments were administered to subjects, who all reported maintenance of their pre-intervention activity levels throughout the study. The study was conducted according to the guidelines of the Declaration of Helsinki, and approved by the Institutional Review Board at the Ohio State University (2017H0395, approved 14 January 2021). Initially, 14 subjects were matched and randomly assigned to the KD + KS and KD + PL groups. In the KD + KS group, one participant declined to participate after enrollment, and another discontinued in the first week due to stomach distress. In the KD + PL group, one participant discontinued due to personal reasons. The group of 12 participants enrolled in the LFD all completed the study. Among those completing each diet (KD + KS *n* = 12; KD + PL *n* = 13; LFD *n* = 12), there were no significant differences in baseline characteristics between groups (Table 1).

### 2.3. Diet Intervention

All food for this controlled hypocaloric feeding study was prepared in a metabolic kitchen. Each meal was prepared and provided to the participants with strict instructions to avoid all other foods outside of non-caloric products. Participants were instructed not to consume artificially sweetened beverages but were allowed to consume other non-caloric products like tea and coffee. Dietitians and research staff developed 7-day rotational meal plans to ensure the highest possible compliance and adherence. Each ingredient was precisely weighed (±0.1 g) with custom macro- and micronutrient composition personalized to each participant using advanced nutrient analysis software (Nutritionist Pro, Axxya Systems, Redmond, WA, USA). Both KD + KS and KD + PL diets were designed based on previous well-formulated standards [21] that includes emphasis on whole foods rich in saturated fat, while the LFD was developed according to United States Department of Agriculture (USDA) Dietary Guidelines for Americans 2015–2020 [30]. Participant food menus were created using a base caloric level of 2000 kcal and then scaled to match 75% of the individual participants estimated energy expenditure based on resting energy expenditure measured by indirect calorimetry (ParvoMedics TrueOne, Salt Lake City, UT, USA, v2400), a standard Harris–Benedict equation for estimating energy expenditure, and the energy cost of their physical activity. All participants were instructed to maintain their habitual physical activity levels throughout the intervention, which ranged among subjects from sedentary to various forms of endurance and resistance training. All diets were isonitrogenous with protein provided at 1.5 g/kg reference weight for all participants, a portion of which was provided as twice daily protein shakes (Metagenics, Inc., Aliso Viejo, CA, USA) containing whey protein isolate (~15 g/serving) and fat containing high oleic sunflower oil and medium chain triglycerides (MCTs). Subjects in both KD arms consumed additional servings of MCT oil (caprylic and capric acid; Metagenics, Inc.) with their breakfast and afternoon snacks. Average nutrient intake over the 6 weeks for each group is shown in Table 2. Additional details of the meal plans are provided in Supplementary Table S1.

The KD + KS group consumed a ketone supplement twice daily consisting of Beta-hydroxybutyrate (βHB) salts and non-caloric flavoring (Metagenics, Inc.), previously reported to induce acute ketosis [31]. One serving contained 11.8 g βHB, 1874 mg sodium, 570 mg calcium, and 57 mg magnesium. βHB content was determined to contain a racemic βHB enantiomer mixture of R- βHB and S- βHB. Participants were instructed to mix the ketone salts with at least eight oz. of water. The KD + PL and LFD groups received a calorie-free placebo containing no βHB or minerals. It was identical in both flavor and appearance to protect the double-blind nature of the study for the KD groups.

### 2.4. Testing Battery

All participants reported to the testing facility between 5:00 and 7:00 a.m. for testing assessments at baseline and after the six-week diet intervention. Participants were instructed to arrive fasted (8–12 h), well rested (8–10 h of sleep), and having refrained from intense exercise the prior two days. Urine specific gravity was measured using a light refractometer to ensure hydration (<1.025). If determined dehydrated, the participant was required to drink eight oz. of water and retest, until achieving euhydration. Each group underwent a battery of tests measuring body composition, resting metabolic rate (RMR), and blood biomarkers. The supplement/placebo was ingested immediately following blood draw and capillary finger sticks. Anthropometric assessments included height, body mass, and whole-body composition using dual-energy x-ray absorptiometry (DXA) (GE Lunar DXA, Madison, WI, USA) by a certified technician. Metabolic rate was measured by indirect calorimetry in a dark, quiet, and temperature-controlled room. A clear hood was placed over the subject’s head to collect samples of air from inhalation and exhalation while breathing normally. Subjects rested in a supine position for 20 min, and then gas exchange was measured for 25 min in 15 s intervals. Steady state values reflective of daily RMR and respiratory exchange ratio (RER) to determine substrate use were selected from stable, average RMR and RER recorded during the final 5 min of continuous readings. Venipuncture was performed by a qualified phlebotomist from an arm vein within the antecubital fossa. Blood was centrifuged, and the resultant plasma and serum aliquots were rapidly snap-frozen in liquid nitrogen and stored at −80 °C for subsequent hematology. A serum separator tube (SST) was obtained during each blood collection. The SST was inverted five times immediately after the blood draw and left to rest for 30 min at room temperature. The tube was spun in a temperature-controlled centrifuge (4 °C) at 1200× *g* for 10 min. and promptly shipped on the same day as collection in an appropriate cooler container to Quest Diagnostics (Secaucus, NJ, USA) to perform a metabolic panel (albumin, globulin, albumin/globulin, bilirubin, alkaline phosphatase (ALP), alanine aminotransferase, aspartate aminotransferase (ALT, AST). Metabolic panel and anthropometric results were used to calculate Hepatic Steatosis Index (HSI) for each subject at both timepoints [32]. In addition, each participant performed daily morning finger sticks while fasted to measure capillary glucose and ketones (Precision Xtra, Chicago, IL, USA).

### 2.5. MRI Acquisition

Each participant was imaged at baseline and post-intervention on a 3T MRI scanner (MAGNETOM Prisma Fit, Siemens Healthineers, Erlangen, Germany) at the same location in the early morning, 3–7 days before the baseline and post-intervention testing batteries. Abdominal liver fat scans were performed in each session [21]. The total duration of the testing session averaged 1h. Sequence and scanning parameters are provided in Supplementary Table S2. The VARiable PROjection (VARPRO) pulse sequence [33] was used to acquire 3D volumetric images covering the entire abdominal region in a single breath-hold to measure liver fat. The VARPRO pulse sequence collects the multiple echo time images required for fat/water separation and automatically generates the in-phase, out-phase, water, water percentage, fat, and fat percentage images that were used to measure liver fat fraction.

### 2.6. Hepatic Fat Quantification

A single operator with five years of MRI experience performed all analyses. Quantification of proton density hepatic fat fraction was determined using the fat percentage maps generated by VARPRO [34]. Liver fat quantification used a previously described methodology [35]. Three measurements in each liver segment were averaged over the height of the liver to measure segmental fat fraction. All of the measurements for the 9 segments were averaged to provide a single composite liver fat fraction.

### 2.7. Statistical Analysis

Statistics were calculated using SPSS v25 (SPSS, Inc., Chicago, IL, USA). Assumptions required for the use of a linear statistics approach were met a priori. Baseline measures were compared for differences between groups with one-way analysis of variance (ANOVA). Outcome variables were analyzed for main effects or interactions using a 3 (group) × 2 (time) pre-post ANOVA design with gender and weight loss as covariates. There were technical problems with the MRI scan of one subject in the KD + KS group, so the liver fat data is based on *n* = 36. We performed a subgroup analysis in individuals with baseline liver fat fraction >5%, meeting the definition of NAFLD. This included 4 participants from the KD + KS group, 3 from the KD + PL group and 5 from the LFD group. The KD groups were combined (Ketogenic Combined) in this sub-due to low sample sizes. A 2 × 2 ANOVA design was used to calculate differences between NAFLD subgroups. Fisher’s Least Significant Difference (LSD) correction was applied to all post-hoc interpretations. Correlations were computed using Pearson product-moment correlation coefficients to determine pairwise associations between selected dependent variables. Two-tail α significance was set a priori at *p* ≤ 0.05.

## 3. Results

### 3.1. Weight Loss and Liver Fat Responses

**Figure 1:**
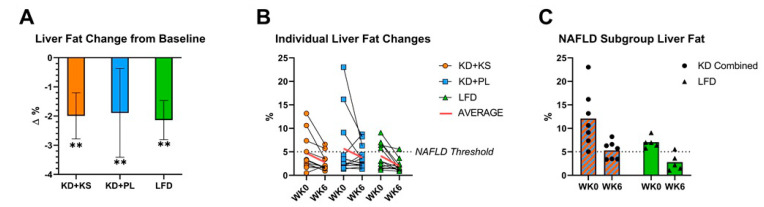
(**A**) Mean and (**B**) individual participant changes in total liver fat after 6-week ketogenic and low-fat diets. (**C**). total liver fat in a subset of participants with nonalcoholic fatty liver disease (NAFLD). ** = *p* < 0.01 from baseline. KD = ketogenic diet. KS = ketone supplement. LFD = low-fat diet WK0 = baseline. WK6 = post-intervention.

Mean weight loss at 6 weeks was significant (*p* < 0.001) in all three groups. There was no significant difference in weight loss between the KD + KS, KD + PL, and LFD groups, representing decreases of 8.1%, 8.5%, and 6.7% of initial body mass, respectively (*p* > 0.05). There were also no differences in changes in whole body fat or lean mass between groups. Composite liver fat content was similar between the KD + KS, KD + PL, and LFD groups at baseline (4.7, 5.7, and 4.1%, respectively; *p* = 0.72). There was a significant (*p* = 0.004) decrease in liver fat post-intervention, but no differences between the KD + KS (−42%), KD + PL (−32%), and LFD (−52%) groups (Figure 1A) (*p* > 0.05). The absolute decrease in percent liver fat was nearly identical between the KD + KS, KD + PL, and LFD groups (−2.0, −1.9, and −2.1%, respectively; *p* = 0.98). There were no significant differences in liver fat response between genders (*p* = 0.40), nor was weight loss a significant covariate for liver fat change (*p* = 0.62).

Despite all subjects having excess adiposity at baseline, liver fat varied considerably, ranging from <1% to 23%. Participants with a liver fat <5% at baseline demonstrated a small variable response post-intervention, but liver fat consistently decreased in those with NAFLD (>5% liver fat) regardless of diet group or the magnitude of weight loss (Figure 1B). Irrespective of diet, in all participants with NAFLD (*n* = 12), there was a marked decrease in liver fat ranging from −13% to −82% (*p* = 0.001). The effect was similar in the Ketogenic Combined (−56%) and LFD (−60%) groups. After the six-week intervention, liver fat fraction dropped below 5% in 7 of the 12 subjects, no longer meeting the definition of NAFLD in those subjects (Figure 1B,C). Analyses of nine liver segments by MRI revealed no significant differences in regional anatomical fat distribution before or after diet is shown in Supplementary Table S3.

### 3.2. Metabolic Responses

**Figure 2:**
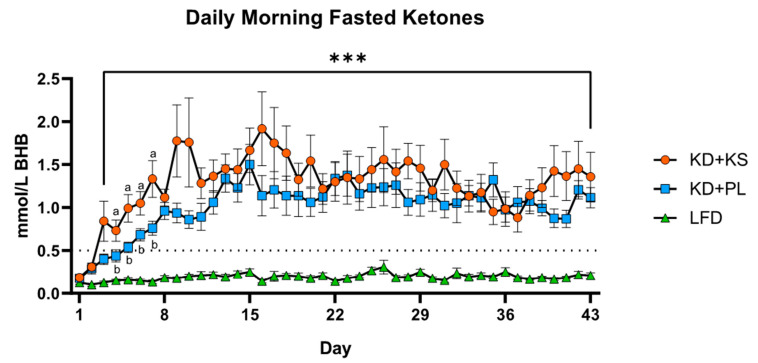
Daily measurements of fasted βHB recorded before breakfast and at least 10 h after supplement ingestion. All values reported as mean ± SEM. KD + KS and KD + PL ketones increased significantly from baseline (***) (*p* < 0.001) and were significantly higher than LFD (*p* < 0.001). Distinct letters denote significant differences between KD + KS and KD + PL.

Metabolic responses are reported for the entire group (Table 3) and the NAFLD subset (Supplementary Table S4). In the entire cohort, fasting capillary βHB increased in all groups and was the only serum marker to show group and interaction effect, as reflected by increased concentrations in the ketogenic groups (Table 3). Hepatic steatosis index (HIS) decreased in the entire cohort post-intervention but there were no group differences (Table 3). Overnight fasted βHB (Figure 2) in KD + KS and KD + PL groups rose steadily during the first week and reached the 0.5 mM threshold indicating ketosis by day 3. The two KD groups remained in nutritional ketosis throughout the intervention, with KD + KS blood ketones greater than KD + PL during the first two weeks. Glucose and alkaline phosphatase decreased, whereas albumin increased post-intervention, but without significant group or interaction effects. There were no differences in other major liver function enzymes: AST, ALT, AST/ALT, and bilirubin from baseline to post-intervention. RMR and RER decreased, but the magnitude was greater for RER in the KD groups. The same general pattern was observed in the NAFLD subset (Table 4) with the same time effects and only ketones and RER showing group differences.

### 3.3. Correlations

**Figure 3:**
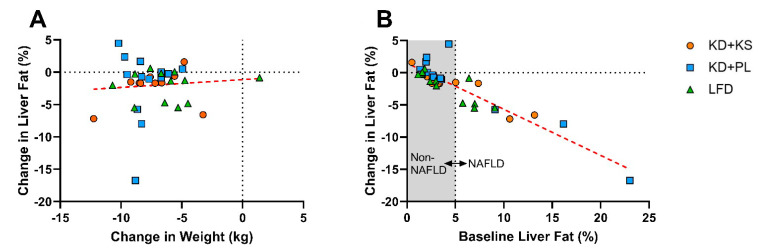
(**A**) Association of the absolute change in liver fat versus weight loss (R = 0.006, *p* > 0.05). (**B**) Association of the absolute change in liver fat versus baseline liver fat (R = −0.91, *p* < 0.05). Within the NAFLD group, the correlation was R = −0.96 for the ketogenic group (*n* = 7) and R = −0.40 for the low-fat diet group (*n* = 5).

The change in liver fat was not correlated with weight loss (Figure 3A) or other changes in anthropometric or circulating markers in the entire cohort and the NAFLD subset as depicted in Supplementary Table S4. The change in liver fat did not correlate with HSI change in the NAFLD subgroup (R = 0.16; *p* = 0.63). The change in liver fat was also not correlated with baseline percent body fat in the full cohort (R = 0.1; *p* = 0.57) or in the NAFLD subgroup (R = −0.04, *p* = 0.90), but it was highly associated with baseline liver fat as demonstrated by consistently greater reductions in those who started with the highest degree of NAFLD (Figure 3B). In KD participants with NAFLD (*n* = 7), 92% of the variability in change in liver fat was explained by baseline liver fat (*p* < 0.001).

## 4. Discussion

The objective of this study was to compare liver fat response following a precisely controlled hypocaloric KD, with and without an exogenous KS, versus an energy-matched LFD in adults with excess adiposity. Notably, the KD included nearly three-fold higher total fat and four-fold higher saturated fat content than the LFD, and yet this did not have any adverse effect liver fat fraction or liver function enzymes. Despite these dramatic differences in macronutrient distribution, when matched for energy intake the experimental diets produced similar weight loss and decrease in liver fat independent of diet composition and ketone supplementation. This cohort of overweight/obese individuals had highly variable liver fat at baseline. The most consistent improvements in liver fat were observed in the one-third of participants classified as having NAFLD (i.e., liver fat > 5%), with smaller fluctuations in individuals with <5% liver fat. Analyses of individual liver segments reflected the overall composite liver fat response. These results emphasize that achieving a consistent caloric deficit can decrease liver fat in the short-term (6 weeks) using different dietary approaches that vary widely in fat, saturated fat, and carbohydrate content.

This study was unique because it compared energy-matched hypocaloric ketogenic and low-fat diets using a controlled feeding design, removing much of the ambiguity about compliance that exists in free-living diet studies that simply counsel subjects on dietary prescriptions. Our finding that hypocaloric low-fat and ketogenic diets resulted in a similar decrease in liver fat after 6-weeks in overweight/obese adults is consistent with results of free-living diet interventions presented by Haufe et al. [18] and Kirk et al. [22], but different than Cunha et al [24] and Browning et al [23]. The most likely explanation for the disparity between our results and those of Cunha [24] is that the KD group lost significantly more weight (−9.7 vs. 1.7 kg) in their study, whereas in other studies when weight loss is similar there is no difference in liver fat responses between a KD and LFD [18,22]. A notable exception is that of Kirk et al [22] who demonstrated a greater decrease in liver fat to a KD versus an LFD after just 2 days (~30 vs. 10%, respectively) under controlled and matched energy conditions, but after 11-weeks when the diets were prescribed in a free-living manner there was no difference between diets. Browning et al. [23] also showed a greater decrease in liver fat to a KD versus an LFD after 2-weeks despite both diets producing equal weight loss. Collectively, these prior data combined with the results of our study suggest that a KD may have a rapid and greater impact on liver fat over the short-term (<2-week) independent of weight loss, but when matched for energy diet composition does not differentially impact liver fat over longer periods of time (2–6 months).

This was the first study to assess liver fat responses to repeated use of an exogenous ketone supplement. Our results showed no additional impact of a ketone salt ingested twice per day beyond that achieved with the KD alone. The dose of ketone salt used elevates ketones approximately 0.5 to 1 mM, but the effect is transient lasting only 1–2 h [30]. We cannot rule out a possible beneficial effect of exogenous ketones in combination with an LFD since we did not test that combination, or a benefit of higher levels of ketones which could be achieved using ketone esters.

While weight loss appears to be an important determinant of liver fat response, the correlation between weight loss (and fat loss) and change in liver fat was weak in our study. A lack of significant association between weight loss and measures of adiposity (whole body fat mass and visceral fat mass) and change in liver fat has been reported before [22,24]). Why the responses in these two fat depots are seemingly disconnected is not well understood but may relate to differential temporal responses with a slight lag in the liver relative to whole body fat loss [11]. One reason may be insufficient variation of weight loss among participants, although it ranged over 10 kg in our study. More likely, the lack of correlation could reflect differences between regulatory factors and temporal responses impacting net loss of body fat versus liver fat.

Although liver fat decreased to a similar extent in all diet groups, the metabolic mechanisms contributing to the net loss of liver fat between low-fat and ketogenic diets are vastly different. As we have previously shown [20], a KD increases whole body fatty acid oxidation as reflected by lower RER, while also promoting enhanced hepatic ketogenesis as evidenced by circulating ketones >1 mM. The metabolism of fatty acid-derived acetyl-CoA through ketogenesis is a primary mechanism by which KD promotes decreased liver fat [16]. Our results suggest that upregulation of these pathways in people consuming a KD, with or without exogenous ketone supplements, is clearly sufficient to compensate for the significantly greater intake of fat, especially in the context of a calorically restricted KD.

Although the greater decrease in liver fat in individuals with NAFLD was expected [18], the strength of the correlation between baseline liver fat and the change in liver fat was remarkable, especially among the KD group with NAFLD. A few individuals demonstrated marked decreases (e.g., 10.6% to 3.4%, and 23.0% to 6.3%), highlighting the therapeutic potential of diet alone in managing more advanced stages of fatty liver. Although baseline liver fat was an excellent predictor of change in liver fat, baseline percent body fat was not. This likely reflects the fact that not all people who develop obesity have fatty liver and/or different factors governing the time course and magnitude of fat accumulation/dissipation in the liver versus the whole body.

An analysis of the anatomical distribution of fat throughout the liver has not been done in prior ketogenic diet studies. The Couinauds classification describes liver anatomy such that each segment has functionally independent vascular flow [36] and therefore potential for variation in nutrient flow and fat content between segments [37,38,39,40]. Similar to previous reports [40], we observed significantly greater anatomical variation in fat throughout the liver in individuals with higher liver fat, but there was a relatively uniform loss of liver fat across segments. Thus, the net hepatic lipid response to hypocaloric diets varying in macronutrients appears to be fairly evenly distributed throughout the entire liver.

Increased consumption of saturated fat in the context of low-carbohydrate intake did not adversely affect enzymes associated with liver health and function, and even marginally improved similar to what other hypocaloric interventions have reported [18]. ALT and AST, the main liver enzymatic markers used for clinical referral for an NAFLD diagnostic exam, trended lower after the diet interventions but did not reach statistical significance. While liver enzymes and tools like the HSI are commonly used as biomarkers of NAFLD, they lack specificity and sensitivity [41,42]. The small but significant increase in albumin and consistent decrease in alkaline phosphatase (ALP) that we observed may provide some level of hepatoprotection as hypoalbuminemia is associated with liver fibrosis and NASH [43,44], and ALP is commonly elevated in fatty liver disease [45,46]. Moreover, the small increase in albumin, attributed mostly to the KD diet groups, could reflect increased protein intake among participants as a result of the properly formulated experimental diets.

One-third of the participants in this study were classified as having NAFLD (>5% liver fat), although none of them had prior knowledge of liver disease. Due to the progressive nature of the disease, early diagnosis is critical [47]. However, diagnosing NAFLD is challenging since the condition is often asymptomatic and the gold standard of diagnosis is liver biopsy, which is prone to sampling error and as an invasive test is not appropriate as a screening tool or to track disease progression [48]. As such, referral for diagnostic testing is usually based on elevated AST and ALT levels, indicative of liver damage. However, there is considerable disagreement regarding unhealthy ranges of AST and ALT, and whether these should be used as proxy measures at all due to lack of sensitivity in detection of liver disease [41,49,50]. Similar to other reports [51], in our NAFLD subgroup only half of the subjects had elevated ALT levels (men > 40 U/L, women > 30 U/L) likely due to the lack of functional damage in the early stages of NAFLD. While MRI is viewed as too costly for screening, the single breath-hold, abdominal fat quantification test that we utilized can be completed in approximately ten minutes, potentially offering a cost-effective approach to diagnosis in high-risk individuals.

A limitation of this study is that we did not acquire liver biopsies to examine liver fibrosis or histology, which would have provided more definitive evidence and severity of NAFLD in those subjects with high liver fat. Over half of the participants with NAFLD (7 out of 12) at baseline no longer met this diagnosis after 6-weeks, likely representing a clinically relevant response; however, agreement on what represents a clinically meaningful change in liver fat is lacking. The absolute decrease in liver fat in NAFLD patients was 5.7% on average, which is likely of clinical benefit as histological improvement has been noted with as little as a 4% decrease [52]. Our ability to detect small differences in liver fat responses between KD and LFD was enhanced by our controlled feeding design but compromised by the relatively small sample size, especially those with NAFLD, and the fact the KDs and the LFD were not randomized. Our enrollment of individuals with excess adiposity, but not specifically fatty liver, likely resulted in a “less responsive” cohort than if we had targeted a group with more advanced, adverse liver conditions, such as NAFLD. The diet interventions lasted just six weeks and involved meticulously prepared meal plans, which may not represent the way people eat in a free-living environment over longer periods of time where maintenance of weight loss is challenging. In our controlled short-term study, dietary macronutrient composition was not a significant factor on hepatic health, whereas in a free-living setting over longer periods of time it may be important [24] due to factors such as diet acceptance and sustainability. We studied liver fat responses in the context of hypocaloric diets that produced significant weight loss. Results may have differed if we tested eucaloric diets that were higher in fat designed to maintain weight. We studied a specific KD that emphasized saturated fat as the primary fat source, whereas people may choose to focus on monounsaturated fat in a free-living setting. Finally, we did not rigorously control for physical activity during the intervention which may have introduced variation to the liver fat responses.

In conclusion, hypocaloric low-fat and high-fat (including saturated fat) KDs (alone or with exogenous ketone supplements) prescribed for 6 weeks produced no significant negative hepatic effects in a group of individuals who were overweight and obese with variable levels of liver fat. In individuals with NAFLD, both an LFD and KD were equally effective at decreasing liver fat at the whole liver level, as well as segmentally. These results indicate that, in the short-term, high saturated fat consumption in the context of low carbohydrate intake does not adversely impact liver health. Moreover, dietary effects on liver health are more dependent on caloric restriction than macronutrient distribution or level of ketosis over several weeks. Practically, this means that a variety of hypocaloric diet paradigms may have a therapeutic effect to decrease liver fat in the context of moderate (5–10%) weight loss under the supervision of a nutrition specialist.

## Figures and Tables

**Table 1:** Participant characteristics.

Baseline Characteristic	KD + KS (*n* = 12)			KD + PL (*n* = 13)			LFD (*n* = 12)			*p*-Value
Sex (male/female)	6/6			6/7			6/6			
Age (years)	35	±	3	35	±	3	35	±	3	0.99
Weight (kg)	90.4	±	3.4	94.1	±	3.2	92.4	±	3.4	0.73
BMI (kg/m^2^)	30.6	±	0.7	31.8	±	0.7	30.9	±	0.7	0.50
PAQ (mets*min/week)	1182	±	269	1168	±	268	1167	±	208	0.99
DXA FM (kg)	31.1	±	1.5	34.5	±	2.3	33.4	±	2.4	0.52
Baseline Liver Fat (%)	4.7	±	3.8	5.5	±	6.2	4.1	±	2.6	0.72
Capillary Ketones (mmol/L βHB)	0.18	±	0.03	0.18	±	0.04	0.13	±	0.02	0.40

Values reported as mean ± SEM. *p*-value obtained from one-way ANOVA (Analysis of variance). BMI = body mass index; PAQ = physical activity questionnaires; DXA = dual-energy x-ray absorptiometry; FM = fat mass; LM = lean mass; βHB = beta-hydroxybutyrate.

**Table 2:** Diet composition.

Nutrient	KD + KS			KD + PL			LFD		
Energy (kcal)	1845	±	102	1752	±	98	1900	±	102
Protein (g)	99	±	3	100	±	3	100	±	3
Carbohydrate (g)	40	±	8 ^a^	38	±	7 ^a^	259	±	8 ^b^
Sugar (g)	17	±	3 ^a^	17	±	3 ^a^	101	±	3 ^b^
Fiber (g)	10	±	1 ^a^	10	±	1 ^a^	34	±	1 ^b^
Added Sugars (g)							<25 g/day		
Fat (g)	143	±	9 ^a^	131	±	8 ^a^	51	±	9 ^b^
SFA (g)	63	±	4 ^a^	63	±	4 ^a^	17	±	4 ^b^
MUFA (g)	38	±	3 ^a^	38	±	3 ^a^	10	±	3 ^b^
PUFA (g)	8	±	1	8	±	1	7	±	1
Cholesterol (g)	414	±	27 ^a^	402	±	26 ^a^	154	±	27 ^b^
Sodium (mg)	6100	±	32 ^a^	2351	±	30 ^b^	1974	±	31 ^c^
Potassium (mg)	2211	±	73 ^a^	2243	±	75 ^a^	2758	±	78 ^b^
Calcium (mg)	2001	±	36 ^a^	880	±	34 ^b^	1008	±	35 ^c^

Values reported as mean ± SEM. Distinct letters denote group differences (*p* < 0.05). SFA = saturated fatty acids; MUFA = monounsaturated fatty acids; PUFA =polyunsaturated fatty acids.

**Table 3:** Serum biomarkers and anthropometric changes.

		Timepoint						3 × 2 ANOVA Effects		
Category	Diet	WK0			WK6			Time	Group	Group* Time
Serum Biomarkers										
AST (U/L)	KD + KS	19.7	±	1.40	18.0	±	1.63	0.32	0.26	0.70
	KD + PL	18.9	±	1.67	18.8	±	1.75			
	LFD	24.2	±	1.75	21.3	±	1.73			
ALT (U/L)	KD + KS	23.2	±	3.30	21.3	±	2.90	0.72	0.74	0.49
	KD + PL	21.0	±	3.67	22.5	±	3.41			
	LFD	28.3	±	4.51	20.9	±	2.06			
AST/ALT Ratio	KD + KS	0.96	±	0.08	0.94	±	0.08	0.51	0.30	0.40
	KD + PL	1.07	±	0.11	0.96	±	0.11			
	LFD	0.99	±	0.09	1.10	±	0.11			
HOMA-IR	KD + KS	2.58	±	0.59	1.45	±	0.26	**<0.001** *	0.45	0.99
	KD + PL	2.68	±	0.58	1.42	±	0.29			
	LFD	3.15	±	0.39	1.96	±	0.40			
Bilirubin (U/L)	KD + KS	0.54	±	0.06	0.55	±	0.06	0.39	0.45	0.54
	KD + PL	0.73	±	0.16	0.71	±	0.18			
	LFD	0.64	±	0.07	0.78	±	0.12			
ALP (U/L)	KD + KS	60.8	±	3.52	48.4	±	2.38	**<0.001** *	0.55	0.21
	KD + PL	63.3	±	7.30	55.6	±	4.64			
	LFD	62.7	±	5.48	57.5	±	5.37			
Albumin (mg/dL)	KD + KS	4.38	±	0.06	4.49	±	0.09	**0.044** *	0.16	0.15
	KD + PL	4.41	±	0.09	4.59	±	0.11			
	LFD	4.57	±	0.09	4.55	±	0.10			
Glucose (mg/dL)	KD + KS	94.0	±	6.00	88.0	±	3.0	**0.035** *	0.87	0.16
	KD + PL	99.0	±	4.00	84.0	±	4.0			
	LFD	92.0	±	3.00	93.0	±	2.0			
Ketones (mmol/L BHB)	KD + KS	0.18	±	0.03	1.36	±	0.28 ^a^	**<0.001** *	**<0.001** *	**<0.001** *
	KD + PL	0.18	±	0.04	1.12	±	0.12 ^a^			
	LFD	0.13	±	0.02	0.21	±	0.03 ^b^			
HSI	KD + KS	39.5	±	4.00	36.7	±	3.23	**<0.001** *	0.64	**0.04** *
	KD + PL	41.5	±	5.30	37.9	±	5.77			
	LFD	39.5	±	3.18	38.6	±	3.78			
Anthropometry										
Weight (kg)	KD + KS	90.4	±	3.7	83.1	±	3.2	**<0.001** *	0.46	0.21
	KD + PL	94.1	±	2.8	86.1	±	2.8			
	LFD	92.4	±	3.4	86.3	±	3.8			
DXA FM (kg)	KD + KS	31.1	±	1.5	26.4	±	1.4	**<0.001** *	0.94	0.34
	KD + PL	34.5	±	2.3	30.2	±	2.2			
	LFD	33.4	±	2.4	29.2	±	2.5			
RMR (kcal/day)	KD + KS	1885	±	120	1653	±	74	**<0.001** *	0.59	0.36
	KD + PL	1739	±	94	1604	±	81			
	LFD	1704	±	44	1591	±	102			
RER (VCO2/VO2)	KD + KS	0.83	±	0.01	0.77	±	0.01 ^a^	**<0.001** *	**<0.001** *	0.68
	KD + PL	0.86	±	0.02	0.78	±	0.01 ^a^			
	LFD	0.88	±	0.02	0.85	±	0.01 ^b^			

Values reported as mean ± SEM. * = *p* < 0.05 (indicated by bold). Distinct letters denote between-group differences (*p*-value < 0.05). AST = aspartate aminotransferase; ALT = alanine aminotransferase; ALP = alkaline phosphatase; HOMA-IR = HOMeostatic Assesment model of Insulin Resistance; DXA = dual-energy x-ray absorptiometry; RMR = resting metabolic rate; RER = respiratory exchange ratio.

**Table 4:** Serum biomarkers and anthropometry changes—NAFLD subgroup (KD *n* = 7; LFD *n* = 5).

		Timepoint						3 × 2 ANOVA Effects		
Category	Diet	WK0			WK6			Time	Group	Group* Time
Serum Biomarkers										
AST (U/L)	KD	21.4	±	1.60	17.9	±	1.87	0.10	**0.024** *	0.71
	LFD	27.6	±	2.16	22.8	±	1.93			
ALT (U/L)	KD	34.3	±	4.68	24.0	±	2.96	0.06	0.33	0.55
	LFD	40.4	±	6.95	25.4	±	2.93			
AST/ALT Ratio	KD	0.70	±	0.12	0.77	±	0.08	0.26	0.57	0.45
	LFD	0.73	±	0.08	0.93	±	0.11			
HOMA-IR	KD	4.56	±	0.90	1.48	±	0.54	**0.03** *	0.94	0.21
	LFD	3.45	±	1.06	2.46	±	0.64			
Bilirubin (U/L)	KD	0.51	±	0.06	0.49	±	0.06	0.16	0.10	0.06
	LFD	0.64	±	0.07	0.98	±	0.25			
ALP (U/L)	KD	61.4	±	6.1	52.3	±	3.60	**0.01** *	0.25	0.98
	LFD	76.6	±	10.0	66.6	±	10.7			
Albumin (mg/dL)	KD	4.44	±	0.12	4.53	±	0.07	0.53	0.42	0.86
	LFD	4.64	±	0.18	4.70	±	0.21			
Glucose (mg/dL)	KD	97.3	±	6.2	84.7	±	2.81	0.35	0.45	0.21
	LFD	84.8	±	6.6	86.2	±	3.34			
Ketones (mmol/L BHB)	KD	0.36	±	0.12	1.59	±	0.33	**0.002** *	**0.02** *	**0.004** *
	LFD	0.09	±	0.01	0.17	±	0.02			
HSI	KD	37.9	±	4.90	35.8	±	3.50	0.20	0.56	0.07
	LFD	37.8	±	1.80	38.2	±	0.02			
Anthropometry										
Weight (kg)	KD	98.3	±	3.6	89.9	±	2.66	<0.001 *	0.78	0.10
	LFD	94.4	±	7.8	89.7	±	8.84			
DXA FM (kg)	KD	75.1	±	3.8	65.0	±	3.48	**<0.001** *	0.66	0.20
	LFD	79.3	±	8.2	71.8	±	9.02			
RMR (kcal/day)	KD	2068	±	111	1790	±	119	0.16	0.23	0.39
	LFD	1789	±	132	1719	±	140			
RER (VCO2/VO2)	KD	0.85	±	0.02	0.76	±	0.01	**0.02** *	**0.02** *	**0.03** *
	LFD	0.87	±	0.02	0.85	±	0.01			

Values reported as mean ± SEM. * = *p* < 0.05 (indicated in bold). DXA = dual-energy x-ray absorptiometry; HSI, hepatic steatosis index; RMR = resting metabolic rate; RER = respiratory exchange ratio.

## Data Availability

Data described in the manuscript will be made available upon reasonable request from.

## Supplementary Materials

The following are available online at https://www.mdpi.com/2072-6643/13/3/966/s1, Table S1: Example daily meal plan; Table S2: MRI sequence parameters; Table S3: Liver lobe fat % distribution and change; Table S4: Pearson Correlations between Liver Fat and Serum Biomarkers/Anthropometric Changes in combined NAFLD subgroup (*n* = 12).

## Abbreviations

ALT, alanine aminotransferase; ANOVA, analysis of variance; ALP, alkaline phosphatase; AST, aspartate aminotransferase; βHB, β-hydroxybutyrate; BMI, body mass index; CV, coefficient of variation; DNL, de novo lipogenesis; DXA, dual-energy x-ray absorptiometry; HSI, hepatic steatosis index; KD, ketogenic diet; KS, ketone supplementation; LSD, least significant difference; LFD, low-fat diet; MRI, magnetic resonance imaging; MCT, medium chain triglycerides; METs, metabolic equivalent; NAFLD, non-alcoholic fatty liver disease; ROI, region of interest; RER, Respiratory Exchange Ratio; RMR, resting metabolic rate; SEM, standard error of mean;, SST, serum separator tube; USDA, United State Department of Agriculture; VARPRO, VARiable PROjection; VLDL, very low-density lipoprotein.

## References

[1] Fernandes J.C. (2017). The GBD 2015 Obesity Collaborators. Health Effects of Overweight and Obesity in 195 Countries over 25 Years. N. Engl. J. Med..

[2] Finkelstein E.A., Khavjou O.A., Thompson H., Trogdon J.G., Pan L., Sherry B., Dietz W. (2012). Obesity and Severe Obesity Forecasts Through 2030. Am. J. Prev. Med..

[3] Formiguera X., Cantón A. (2004). Obesity: Epidemiology and clinical aspects. Best Pr. Res. Clin. Gastroenterol..

[4] Johnstone A.M., Horgan G.W., Murison S.D., Bremner D.M., Lobley G.E. (2008). Effects of a high-protein ketogenic diet on hunger, appetite, and weight loss in obese men feeding ad libitum. Am. J. Clin. Nutr..

[5] A Parry S., Hodson L. (2017). Influence of dietary macronutrients on liver fat accumulation and metabolism. J. Investig. Med..

[6] Must A., Strauss R. (1999). Risks and consequences of childhood and adolescent obesity. Int. J. Obes..

[7] Luukkonen P.K., Sädevirta S., Zhou Y., Kayser B., Ali A., Ahonen L., Lallukka S., Pelloux V., Gaggini M., Jian C. (2018). Saturated Fat Is More Metabolically Harmful for the Human Liver Than Unsaturated Fat or Simple Sugars. Diabetes Care.

[8] Garbow J.R., Doherty J.M., Schugar R.C., Travers S., Weber M.L., Wentz A.E., Ezenwajiaku N., Cotter D.G., Brunt E.M., Crawford P.A. (2011). Hepatic steatosis, inflammation, and ER stress in mice maintained long term on a very low-carbohydrate ketogenic diet. Am. J. Physiol. Liver Physiol..

[9] Zhang X., Qin J., Zhao Y., Shi J., Lan R., Gan Y., Ren H., Zhu B., Qian M., Du B. (2016). Long-term ketogenic diet contributes to glycemic control but promotes lipid accumulation and hepatic steatosis in type 2 diabetic mice. Nutr. Res..

[10] Douris N., Melman T., Pecherer J.M., Pissios P., Flier J.S., Cantley L.C., Locasale J.W., Maratos-Flier E. (2015). Adaptive changes in amino acid metabolism permit normal longevity in mice consuming a low-carbohydrate ketogenic diet. Biochim. Biophys. Acta (BBA) Mol. Basis Dis..

[11] Watanabe M., Singhal G., Fisher F.M., Beck T.C., Morgan D.A., Socciarelli F., Mather M.L., Risi R., Bourke J., Rahmouni K. (2019). Liver-derived FGF21 is essential for full adaptation to ketogenic diet but does not regulate glucose homeostasis. Endocr..

[12] Kang H.C., Chung D.E., Kim D.W., Kim H.D. (2004). Early- and Late-onset Complications of the Ketogenic Diet for Intractable Epilepsy. Epilepsia.

[13] Stevens C.E., Turner Z., Kossoff E.H. (2016). Hepatic Dysfunction as a Complication of Combined Valproate and Ketogenic Diet. Pediatr. Neurol..

[14] Mardinoglu A., Wu H., Bjornson E., Zhang C., Hakkarainen A., Räsänen S.M., Lee S., Mancina R.M., Bergentall M., Pietiläinen K.H. (2018). An Integrated Understanding of the Rapid Metabolic Benefits of a Carbohydrate-Restricted Diet on Hepatic Steatosis in Humans. Cell Metab..

[15] Donnelly K.L., Smith C.I., Schwarzenberg S.J., Jessurun J., Boldt M.D., Parks E.J. (2005). Sources of fatty acids stored in liver and secreted via lipoproteins in patients with nonalcoholic fatty liver disease. J. Clin. Investig..

[16] Luukkonen P.K., Dufour S., Lyu K., Zhang X.-M., Hakkarainen A., Lehtimäki T.E., Cline G.W., Petersen K.F., Shulman G.I., Yki-Järvinen H. (2020). Effect of a ketogenic diet on hepatic steatosis and hepatic mitochondrial metabolism in nonalcoholic fatty liver disease. Proc. Natl. Acad. Sci. USA.

[17] Yki-Järvinen H. (2015). Nutritional Modulation of Non-Alcoholic Fatty Liver Disease and Insulin Resistance. Nutrients.

[18] Haufe S., Engeli S., Kast P., Böhnke J., Utz W., Haas V., Hermsdorf M., Mähler A., Wiesner S., Birkenfeld A.L. (2011). Randomized comparison of reduced fat and reduced carbohydrate hypocaloric diets on intrahepatic fat in overweight and obese human subjects. Hepatology.

[19] Tendler D., Lin S., Yancy W.S., Mavropoulos J., Sylvestre P., Rockey D.C., Westman E.C. (2007). The Effect of a Low-Carbohydrate, Ketogenic Diet on Nonalcoholic Fatty Liver Disease: A Pilot Study. Dig. Dis. Sci..

[20] A LaFountain R., Miller V.J., Barnhart E.C., Hyde P.N., Crabtree C.D., McSwiney F.T., Beeler M.K., Buga A., Sapper T.N., A Short J. (2019). Extended Ketogenic Diet and Physical Training Intervention in Military Personnel. Mil. Med..

[21] Hyde P.N., Sapper T.N., Crabtree C.D., LaFountain R.A., Bowling M.L., Buga A., Fell B., McSwiney F.T., Dickerson R.M., Miller V.J. (2019). Dietary carbohydrate restriction improves metabolic syndrome independent of weight loss. JCI Insight.

[22] Kirk E., Reeds D.N., Finck B.N., Mayurranjan M.S., Patterson B.W., Klein S. (2009). Dietary Fat and Carbohydrates Differentially Alter Insulin Sensitivity During Caloric Restriction. Gastroenterol..

[23] Browning J.D., A Baker J., Rogers T., Davis J., Satapati S., Burgess S.C. (2011). Short-term weight loss and hepatic triglyceride reduction: Evidence of a metabolic advantage with dietary carbohydrate restriction. Am. J. Clin. Nutr..

[24] Cunha G., De Mello L.L.C., Hasenstab K., Spina L., Bussade I., Mesiano J.M.P., Coutinho W., Guzman G., Sajoux I. (2020). MRI estimated changes in visceral adipose tissue and liver fat fraction in patients with obesity during a very low-calorie-ketogenic diet compared to a standard low-calorie diet. Clin. Radiol..

[25] Watanabe M., Tozzi R., Risi R., Tuccinardi D., Mariani S., Basciani S., Spera G., Lubrano C., Gnessi L. (2020). Beneficial effects of the ketogenic diet on nonalcoholic fatty liver disease: A comprehensive review of the literature. Obes. Rev..

[26] Stubbs B.J., Cox P.J., Evans R.D., Santer P., Miller J.J., Faull O.K., Magor-Elliott S., Hiyama S., Stirling M., Clarke K. (2017). On the Metabolism of Exogenous Ketones in Humans. Front. Physiol..

[27] Egan B. (2018). The glucose-lowering effects of exogenous ketones: Is there therapeutic potential?. J. Physiol..

[28] Idilman I.S., Aniktar H., Idilman R., Kabacam G., Savas B., Elhan A., Celik A., Bahar K., Karcaaltincaba M. (2013). Hepatic Steatosis: Quantification by Proton Density Fat Fraction with MR Imaging versus Liver Biopsy. Radiology.

[29] Buga A., Kackley M.L., Crabtree C.D., Sapper T.N., McCabe L., Fell B., LaFountain R.A., Hyde P.N., Martini E.R., Bowman J. (2021). The Effects of a Six-Week Controlled, Hypocaloric Ketogenic Diet, with and without Exogenous Ketone Salts, on Body Composition Responses. Front. Nutr..

[30] US Department of Agriculture and Department of Health and Human Services (2015). 2015–2020 Dietary Guidelines for Americans.

[31] O’Connor A., Chang J.-L., Brownlow M., Contractor N. (2018). Acute oral intake of beta-hydroxybutyrate in a pilot study transiently increased its capillary levels in healthy volunteers. J. Nutr. Heal. Food Eng..

[32] Chen L.-M., Huang J.-F., Chen Q.-S., Lin G.-F., Zeng H.-X., Lin X.-F., Lin X.-J., Lin L., Lin Q.-C. (2019). Validation of fatty liver index and hepatic steatosis index for screening of non-alcoholic fatty liver disease in adults with obstructive sleep apnea hypopnea syndrome. Chin. Med. J..

[33] Hernando D., Haldar J.P., Sutton B.P., Ma J., Kellman P., Liang Z.-P. (2008). Joint estimation of water/fat images and field inhomogeneity map. Magn. Reson. Med..

[34] Springer F. (2010). Liver fat content determined by magnetic resonance imaging and spectroscopy. World J. Gastroenterol..

[35] Crabtree C.D., LaFountain R.A., Hyde P.N., Chen C., Pan Y., Lamba N., Sapper T.N., Short J.A., Kackley M.L., Buga A. (2019). Quantification of Human Central Adipose Tissue Depots: An Anatomically Matched Comparison Between DXA and MRI. Tomography.

[36] Mitra V., Metcalf J. (2012). Functional anatomy and blood supply of the liver. Anaesth. Intensiv. Care Med..

[37] Dulai P.S., Sirlin C.B., Loomba R. (2016). MRI and MRE for non-invasive quantitative assessment of hepatic steatosis and fibrosis in NAFLD and NASH: Clinical trials to clinical practice. J. Hepatol..

[38] Regnell S.E., Peterson P., Trinh L., Broberg P., Leander P., Lernmark Å, Månsson S., Larsson H.E. (2015). Magnetic resonance imaging reveals altered distribution of hepatic fat in children with type 1 diabetes compared to controls. Metabolism.

[39] Sofue K., Mileto A., Dale B.M., Zhong X., Bashir M.R. (2015). Interexamination repeatability and spatial heterogeneity of liver iron and fat quantification using MRI-based multistep adaptive fitting algorithm. J. Magn. Reson. Imaging.

[40] Bonekamp S., Tang A., Mashhood A., Wolfson T., Bs C.C., Middleton M.S., Clark L., Gamst A., Loomba R., Sirlin C.B. (2014). Spatial distribution of MRI-determined hepatic proton density fat fraction in adults with nonalcoholic fatty liver disease. J. Magn. Reson. Imaging.

[41] Browning J.D., Szczepaniak L.S., Dobbins R., Nuremberg P., Horton J.D., Cohen J.C., Grundy S.M., Hobbs H.H. (2004). Prevalence of hepatic steatosis in an urban population in the United States: Impact of ethnicity. Hepatology.

[42] Angulo P. (2008). Obesity and Nonalcoholic Fatty Liver Disease. Nutr. Rev..

[43] Fierbinteanu-Braticevici C., Baicus C., Tribus L., Papacocea R. (2011). Predictive factors for nonalcoholic steatohepatitis (NASH) in patients with nonalcoholic fatty liver disease (NAFLD). J. Gastrointest. Liver Dis..

[44] Angulo P., Hui J.M., Marchesini G., Bugianesi E., George J., Farrell G.C., Enders F., Saksena S., Burt A.D., Bida J.P. (2007). The NAFLD fibrosis score: A noninvasive system that identifies liver fibrosis in patients with NAFLD. Hepatology.

[45] McCullough A.J. (2004). The clinical features, diagnosis and natural history of nonalcoholic fatty liver disease. Clin. Liver Dis..

[46] Pantsari M.W., Harrison S.A. (2006). Nonalcoholic Fatty Liver Disease Presenting With an Isolated Elevated Alkaline Phosphatase. J. Clin. Gastroenterol..

[47] Ali R., Cusi K. (2009). New diagnostic and treatment approaches in non-alcoholic fatty liver disease (NAFLD). Ann. Med..

[48] Hines C.D., Frydrychowicz A., Hamilton G., Tudorascu D.L., Vigen K.K., Yu H., McKenzie C.A., Sirlin C.B., Brittain J.H., Reeder S.B. (2011). T1 independent, T2* corrected chemical shift based fat-water separation with multi-peak fat spectral modeling is an accurate and precise measure of hepatic steatosis. J. Magn. Reson. Imaging.

[49] Mohamadnejad M., Pourshams A., Malekzadeh R., Mohamadkhani A., Rajabiani A., Asgari A.A., Alimohamadi S.M., Razjooyan H., Mamar-Abadi M. (2003). Healthy ranges of serum alanine aminotransferase levels in Iranian blood donors. World J. Gastroenterol..

[50] Kang H.S., Um S.H., Seo Y.S., An H., Lee K.G., Hyun J.J., Kim E.S., Park S.C., Keum B., Kim J.H. (2010). Healthy range for serum ALT and the clinical significance of “unhealthy” normal ALT levels in the Korean population. J. Gastroenterol. Hepatol..

[51] Clark J.M., Brancati F.L., Diehl A.M. (2003). The prevalence and etiology of elevated aminotransferase levels in the United States. Am. J. Gastroenterol..

[52] Patel J., Bettencourt R., Cui J., Salotti J., Hooker J., Bhatt A., Hernandez C., Nguyen P., Aryafar H., Valasek M. (2016). Association of noninvasive quantitative decline in liver fat content on MRI with histologic response in nonalcoholic steatohepatitis. Ther. Adv. Gastroenterol..

