# Supplementary material for: Comparison of Ketogenic Diets with and without Ketone Salts versus a Low-Fat Diet: Liver Fat Responses in Overweight Adults
Source: Nutrients. 2021 Mar 17;13(3):966. doi: 10.3390/nu13030966 (PMC8002465; doi:10.3390/nu13030966)
Supplement: Supplementary file 1 [file nutrients-13-00966-s001.pdf]

Supplementary Table S1. Example daily meal plan.

Ketogenic Diet	Weight	High Carbohydrate	Weight.
Breakfast (Peanut Butter Cup Shake)		Breakfast (Oatmeal)	
Chocolate Ketogenic shake (fat and protein) (Metagenics, Inc.)	(54.0 g)	Old Fashion oats	(50.0 g)
Heavy cream	(10.0 g)	Chia seed	(3.0 g)
Almond milk	(240.0 g)	Banana	(75.0 g)
Peanut butter	(32.0 g)	Dates	(30.0 g)
MCT oil	(10.0 g)	Peanut butter	(15.0 g)
		Cinnamon, Ground	(1.0 g)
		Raisins	(20.0 g)
		Skim milk	(350.0 g)
Lunch (Dijon Salmon w/ Broccoli)		Lunch (Dijon Salmon w/Veggies)	
Salmon	(110.0 g)	Salmon	(115.0 g)
Olive oil	(7.8 g)	Olive oil	(2.0 g)
Parmesan cheese	(5.0 g)	Parmesan cheese	(5.0 g)
Dijon mustard	(5.0 g)	Dijon mustard	(5.0 g)
Lemon juice	(2.0 g)	Lemon juice	(2.0 g)
Broccoli	(95.0 g)	Broccoli	(95.0 g)
Parmesan cheese	(5.0 g)	Lite butter	(3.0 g)
Butter, Unsalted	(5.7 g)	Mashed sweet potatoes	(180.0 g)
Afternoon Snack		Afternoon Snack	
MCT oil	(10.0 g)	Apple slices	(100.0 g)
Chicken bouillon	(2.5 g)	Popcorn	(18.0 g)
Almonds	(30.0 g)	Vanilla yogurt	(227.0 g)
Sugar free Jello	(96.0 g)		
Cheddar cheese cubes	(43.0 g)		
Dinner (Zoodle Chicken Alfredo)		Dinner (Chicken Fajitas w/ Rice)	
Zucchini noodles	(200.0 g)	Fajita roasted chicken	(99.0 g)
Butter	(5.0 g)	Peppers & onion	(110.0 g)
Heavy cream	(58.0 g)	Sour Cream	(24.0 g)
Cream cheese	(25.0 g)	Salsa	(60.0 g)
Pesto	(10.0 g)	Whole wheat tortilla	(45.0 g)
Parmesan cheese	(5.0 g)	Corn	(60.0 g)
Roasted chicken	(90.0 g)	Black beans & brown rice	(110.0 g)

Supplementary Table S2. MRI sequence parameters.

	Field Strength	Total Slice	Acquisition Time	Repetition Time (TR)	Echo Time (TE)	Flip Angle	Slice Thickness	Slice Coverage	FOV_x	FOV_y
	(T)	(#)	(s)	(ms)	(ms)	(degrees)	(mm)	(mm)	(mm)	(mm)
Fat Imaging (Abdominal)	3 Tesla	64	18	9.06	1.2	4	4.8	307	282–390	418–500

FOV = field of view.

Supplementary Table S3. Liver lobe fat % distribution and change.

		Timepoint						3 × 2 ANOVA Effects		
**Segment**	**Diet**	**WK0**			**WK6**			**Time**	**Group**	**Group*Time**
Superior Lobes										
1	KD + KS	4.75	±	1.10	2.74	±	0.41	**0.005***	0.16	0.73
	KD + PL	5.66	±	1.56	3.69	±	0.59			
	LFD	3.93	±	0.70	1.96	±	0.33			
2	KD + KS	4.52	±	1.10	2.91	±	0.49	**0.016***	0.13	0.65

	KD + PL	5.73	±	1.90	3.85	±	0.64			
	LFD	3.67	±	0.69	2.01	±	0.20			
4a	KD + KS	4.93	±	1.26	1.96	±	0.42	**0.001***	0.23	0.61
	KD + PL	5.53	±	1.65	3.75	±	0.73			
	LFD	3.95	±	0.69	1.89	±	0.49			
8	KD + KS	5.21	±	1.25	2.70	±	0.68	**0.003***	0.33	0.82
	KD + PL	5.96	±	2.01	4.13	±	0.81			
	LFD	4.71	±	0.93	2.16	±	0.55			
7	KD + KS	4.58	±	1.58	3.44	±	0.85	**0.009***	0.39	0.66
	KD + PL	5.76	±	1.97	3.69	±	0.75			
	LFD	4.22	±	0.91	2.08	±	0.44			
Inferior Lobes										
3	KD + KS	4.68	±	1.29	2.89	±	0.62	**0.004***	0.19	0.71
	KD + PL	5.87	±	1.79	3.73	±	0.67			
	LFD	4.08	±	0.74	2.08	±	0.36			
4b	KD + KS	5.01	±	1.43	2.42	±	0.60	**0.005***	0.43	0.88
	KD + PL	5.92	±	1.91	3.74	±	0.64			
	LFD	4.83	±	1.07	2.06	±	0.52			
5	KD + KS	4.71	±	1.04	2.65	±	0.70	**0.011***	0.26	0.78
	KD + PL	5.42	±	2.08	3.93	±	0.89			
	LFD	4.16	±	0.95	1.99	±	0.59			
6	KD + KS	4.34	±	1.31	3.08	±	0.89	**0.007***	0.31	0.80
	KD + PL	5.39	±	1.85	3.70	±	0.75			
	LFD	3.70	±	0.76	1.75	±	0.38			

Values reported as mean ± SEM. * = $p < 0.05$ (indicated in bold).

Supplementary Table S4. Pearson Correlations between Liver Fat and Serum Biomarkers/Anthropometric Changes in combined NAFLD subgroup ($n = 12$).

Category	R	*p*-Value
Serum		
AST (U/L)	−0.055	0.86
ALT (U/L)	0.027	0.93
AST/ALT	−0.004	0.99
HOMA-IR	0.455	0.14
Bilirubin (U/L)	0.075	0.82
ALP (U/L)	0.157	0.63
Albumin (mg/dL)	0.175	0.59
Glucose (mg/dL)	−0.188	0.56
Ketones (mmol/L BHB)	−0.037	0.91
HSI	0.157	0.63
Anthropometry		
Weight (kg)	0.338	0.28
DEXA FM (kg)	0.264	0.41
RMR (kcal/day)	0.321	0.34
RER (VCO2/VO2)	0.165	0.61

Values in bold face and * = $p < 0.05$. AST = aspartate aminotransferase; ALT = alanine aminotransferase; ALP = alkaline phosphatase; HOMA-IR = HOMeostatic Assesment model of Insulin Resistance; DXA = dual-energy x-ray absorptiometry; RMR = resting metabolic rate; RER = respiratory exchange ratio.
